# Radiological features in pediatric myelin oligodendrocyte glycoprotein antibody-associated disease—diagnostic criteria and lesion dynamics

**DOI:** 10.1007/s00247-024-06023-2

**Published:** 2024-09-07

**Authors:** Li-tal Pratt, Hadas Meirson, Mika Shapira Rootman, Liat Ben-Sira, Shelly I Shiran

**Affiliations:** 1 Pediatric Radiology, Imaging Division, Tel Aviv Sourasky Medical Center, 6 Weizmann Street, 6423906 Tel Aviv, Israel; 2 Sackler Faculty of Medicine, Tel Aviv University, Tel Aviv, Israel; 3 Pediatric Neurology Institute, Dana-Dwek Children’s Hospital, Tel Aviv Sourasky Medical Center, Tel Aviv, Israel; 4 The Department of Diagnosing Imaging, Rambam Medical Center, Haifa, Israel

**Keywords:** Acute disseminated encephalomyelitis, Aquaporin 4-IgG-seropositive neuromyelitis optica spectrum disorder, Demyelinating diseases, Magnetic resonance imaging, Multiple sclerosis, Myelin oligodendrocyte glycoprotein antibody-associated disease, Optic neuritis, Transverse myelitis

## Abstract

The spectrum of acquired pediatric demyelinating syndromes has been expanding over the past few years, to include myelin oligodendrocyte glycoprotein antibody-associated disease (MOGAD), as a distinct neuroimmune entity, in addition to pediatric-onset multiple sclerosis (POMS) and aquaporin 4-IgG-seropositive neuromyelitis optica spectrum disorder (AQP4+NMOSD). The 2023 MOGAD diagnostic criteria require supporting clinical or magnetic resonance imaging (MRI) features in patients with low positive myelin oligodendrocyte glycoprotein IgG titers or when the titers are not available, highlighting the diagnostic role of imaging in MOGAD. In this review, we summarize the key diagnostic features in MOGAD, in comparison to POMS and AQP4+NMOSD. We describe the lesion dynamics both during attack and over time. Finally, we propose a guideline on timing of imaging in clinical practice.

## Introduction

The spectrum of acquired demyelinating syndromes has dramatically evolved over the past two decades. The discovery of specific autoantibodies targeted against proteins in the central nervous system (CNS) allowed recognition of inflammatory demyelinating conditions that are distinct from multiple sclerosis [1–3]. In 2004, the identification of aquaporin 4 (AQP4) antibodies directed against the main water channel protein in the CNS [4] led to the recognition of seropositive neuromyelitis optica spectrum disorder (AQP4+NMOSD), in patients previously labelled as optico-spinal multiple sclerosis. The first association between myelin oligodendrocyte glycoprotein (MOG) antibodies and acute disseminated encephalomyelitis (ADEM) was reported in 2007 [5]. Since then, MOG antibodies’ detection techniques have improved [6] and MOG antibodies have been shown to be associated with an evolving group of demyelinating and encephalitic conditions, altogether termed MOG antibody-associated disease (MOGAD), with predilection for the pediatric population and diverse radiological phenotypes [2, 7].

In 2023, the international MOGAD panel established formal consensus criteria for diagnosis of MOGAD as a distinct entity [8]. The criteria require detection of MOG antibodies and a presentation of a core clinical demyelinating event, including ADEM, optic neuritis, transverse myelitis, cerebral cortical encephalitis often with seizures, brainstem or cerebellar deficits, and cerebral monofocal or polyfocal deficits [8], with exclusion of other explanatory diagnoses. In cases with equivocal MOG antibody seropositivity results (low positive, positive without reported titer, or negative but cerebrospinal fluid (CSF) positive), the diagnostic criteria rely on supporting clinical and magnetic resonance imaging (MRI) features that will be discussed. MRI is a key component in the proposed criteria and is essential for diagnosis as well as for follow-up of MOGAD patients, as in recent years there is a growing evidence of unique lesion dynamics during and post MOGAD attacks [9–11].

In this review, we will discuss the various MRI appearances of pediatric MOGAD at presentation of disease and during surveillance, in light of the new proposed diagnostic criteria, and compared to pediatric-onset multiple sclerosis (POMS) and AQP4+NMOSD.

## Background and diagnostic approach

MOG antibodies target a CNS-specific protein that is exclusively expressed on the surface of oligodendrocytes and myelin, and plays a role in the maturation of oligodendrocytes, as well as in maintaining myelin integrity [2, 7]. Although MOG constitutes only a minor component of myelin (0.05%), it is highly immunogenic and may incite the production of autoantibodies [2]. The disease is rare, with higher prevalence in children as compared to adults, and an incidence of approximately 0.3 per 100,000 in children [12]. These numbers may reflect underestimation of true prevalence, due to the wide range of disease presentations, and variable availability of serologic assessment. MOGAD has been identified in up to 40% of pediatric (<18 years) acquired demyelinating disorders [6, 13]. There is an equal prevalence between female and male pediatric patients (1:1), as opposed to female preponderance in POMS (3:1) and AQP4+NMOSD (9:1) [14–16]. The course of disease may be monophasic or relapsing [8, 16], as opposed to chronic relapsing nature characterizing POMS and AQP4+NMOSD. The clinical presentation may be acute or subacute, with symptoms gradually developing over days to weeks, until reaching a nadir. In many cases, an infection or, less commonly, a vaccination precedes the demyelinating event by 1–3 weeks [17, 18].

The diagnostic work-up of a child presenting with a clinically suspected demyelinating event should include clinical, laboratory, and imaging evaluation. Clinical evaluation includes a thorough history and a full physical examination including neurological and neuroophthalmological assessment (with optical coherence tomography (OCT) and visual evoked potentials (VEPs)). Laboratory testing should include serological assessment of MOG-IgG and AQP4-IgG antibodies in the blood by cell-based assay and oligoclonal bands in the blood, as well as CSF sampling for white blood cells, protein, and oligoclonal bands. Neuroimaging includes a contrast-enhanced MRI scan of the brain, orbits, and spine, to assess for multifocality (Table 1), in accordance with the Magnetic Resonance Imaging in Multiple Sclerosis (MAGNIMS) network consensus guidelines [19].

**Table 1:** Magnetic resonance imaging protocol for assessment of pediatric myelin oligodendrocyte glycoprotein antibody-associated disease^a^

	Sequence	Plane	Slice thickness (mm)
Whole brain	T1-weighted 3-D gradient echo	Sagittal (with axial and coronal reconstructions)	1 mm isotropic is preferred but, if over contiguous (through plane and in plane), not >1.5 mm, with 0.75 mm overlap
	T2-weighted 3-D-FLAIR	Sagittal (with axial and coronal reconstructions)	
	DWI	Axial	≤5 mm with a 10–30% gap
Gadolinium contrast injection Standard doses of 0.1 mmol/kg bodyweight, macrocyclic gadolinium chelates only^b^			
Orbits	T2-weighted (TSE or FSE) with fat suppression or STIR	Coronal	≤2–3 mm with no gap
		Axial	
Whole brain	T2-weighted (TSE or FSE)	Axial	≤3 mm with no gap
	SWI	Axial	≤2.5 mm with a 10–30% gap
	T1-weighted 3-D gradient echo	Sagittal (with axial and coronal reconstructions)	1 mm isotropic is preferred but, if over contiguous (through plane and in plane), not >1.5 mm, with 0.75 mm overlap
	T2-weighted FLAIR	Axial	≤3 mm with no gap
Orbits	T1-weighted (TSE or FSE) with fat suppression	Coronal	≤3 mm with no gap
		Axial	
Whole spine	T1-weighted sequences (TSE or FSE)	Sagittal	≤2–3 mm with no gap
		Axial	≤4 mm with no gap
	T2-weighted (TSE or FSE)	Sagittal	≤3 mm with no gap
		Axial	≤4 mm with no gap
	T2-weighted STIR	Sagittal	≤3 mm with no gap

^a^The suggested protocol is consistent with the Magnetic Resonance Imaging in Multiple Sclerosis (MAGNIMS) network consensus guidelines [19], with modifications that are based on this review of the literature, that include acquisition of whole brain, orbits, and spine to assess for multifocality on initial presentation. Sequences of the spine include the lumbar spine for assessment of nerve root enhancement in the cauda equina. Dedicated fat-saturated sequences of the orbits are required for evaluation of inflammatory changes along the optic nerves and perineural tissues

^b^The interval between contrast injection and T1-weighted acquisition should be at least 5–10 min, and should be consistent on follow-up scans. This protocol is suggested for initial diagnosis and for the first follow-up scan (3 months after initial presentation), which is performed to establish a new baseline accounting for radiologic lag changes. Additional follow-up studies should be tailored to the child’s clinical and treatment status. Given the evidence regarding gadolinium-deposition in the brain, contrast-enhanced sequences are not routinely acquired on follow-up scans, unless clinically indicated for treatment decision

*D* dimensional, *DWI* diffusion-weighted imaging, *FLAIR* fluid-attenuated inversion recovery, *FSE* fast spin echo, *STIR* short tau inversion recovery, *SWI* susceptibility weighted imaging, *TSE* turbo spin echo

## Clinical-radiological phenotypes at presentation

There is a wide variety of clinical-MRI phenotypes associated with MOGAD. The initially described patterns include ADEM, optic neuritis, and transverse myelitis. These can occur separately or in combination, altogether comprising more than 90% of pediatric MOGAD presentations [20–22]. Included are also children with neuromyelitis optica spectrum disorder-like phenotype that can present with simultaneous or sequential optic neuritis and transverse myelitis, and can exhibit other core features of neuromyelitis optica spectrum disorder such as brainstem syndrome. MOG antibodies are common in AQP4-IgG-seronegative neuromyelitis optica spectrum disorder, being reported in up to 83.4% of pediatric AQP4-IgG-seronegative patients [23, 24].

Intra-attack asymptomatic lesions can be observed in the brain, optic nerves, and spinal cord – for example, asymptomatic ADEM-like brain lesions, in a child presenting with transverse myelitis or optic neuritis. Intra-attack asymptomatic brain and optic nerve lesions have been detected in 33–50% of patients with MOGAD transverse myelitis [8], further emphasizing the importance of scanning the entire neuroaxis [15].

Over the last decade, the MOGAD umbrella has been continuously expanding to include other recognized clinical or radiological brain patterns that are not compatible with the definition of ADEM, according to the International Pediatric Multiple Sclerosis Study Group (IPMSSG) [25]. These comprise autoimmune cortical encephalitis [26], brainstem and/or cerebellar syndromes [27], leptomeningeal enhancement [28], and cerebral monofocal or polyfocal CNS deficits associated with demyelinating lesions.

Other rare presentations have been reported, mostly in adult case reports and small series, including cranial neuropathies and concomitant peripheral patterns/combined central and peripheral demyelination [8, 22, 29–31].

The MOGAD phenotype is age dependent, which might reflect variability in MOG expression at different age groups. Young children (<11 years) tend to present with ADEM phenotype, while older patients (≥11 years) present more commonly with optic neuritis [14, 20]. The severity of attacks and degree of recovery are also age dependent, with younger children presenting with worst clinical-radiological severity, but with faster and more complete recovery [6].

In the following subsections, MOGAD imaging findings will be described according to the involved neuroanatomic structure, and with reference to the newly diagnostic MOGAD criteria.

### Brain involvement

Lesions in the brain can be associated with MOGAD ADEM, neuromyelitis optica spectrum disorder-like phenotype, autoimmune encephalitis, or brainstem/cerebellar syndromes.

#### MOGAD ADEM

ADEM is an encephalopathy associated with multifocal neurologic deficits (motor deficits, seizures, and cerebellar symptoms), as defined by the IPMSSG [25]. Fifty percent of children presenting with a first ADEM attack will have MOG antibodies [8, 21]. In addition, almost all patients demonstrating a relapsing course of disease, namely multiphasic disseminated encephalomyelitis or ADEM followed by optic neuritis (ADEM-optic neuritis), will be MOG antibody seropositive [13, 32].

**Fig. 1:**
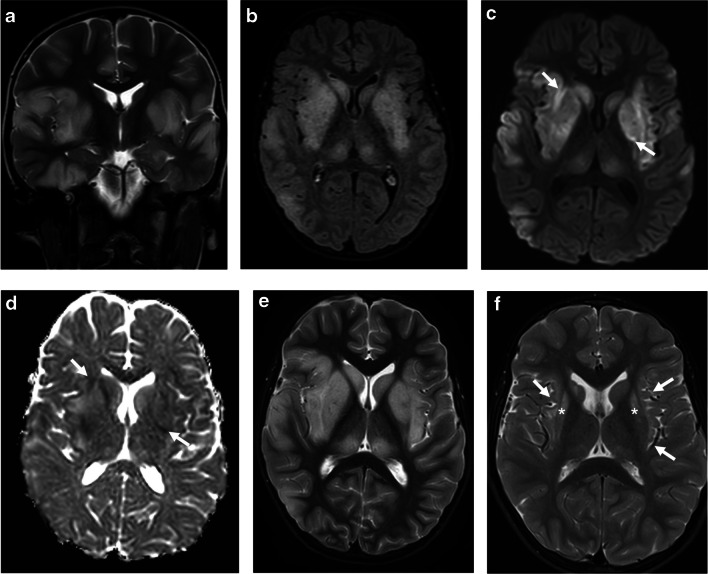
Acute disseminated encephalomyelitis pattern involving the brain in a 6-year-old boy with myelin oligodendrocyte glycoprotein antibody seropositivity: **a**–**d** coronal T2-weighted (**a**), axial fat-suppressed fluid-attenuated inversion recovery (FLAIR) (**b**), axial diffusion-weighted (*b*=1,000) (**c**), and corresponding apparent diffusion coefficient map (**d**) magnetic resonance (MR) images at initial presentation show confluent, extensive, and “fluffy” increased T2 and FLAIR signal changes involving the basal ganglia, thalami, bilateral cortices, and juxta-cortical white matter, mostly affecting the temporo-occipital regions. Few areas demonstrate diffusion restriction (*arrows* in **c**, **d**). **e**, **f** Axial T2-weighted MR images at initial presentation (**e**) and at 2-year follow-up (**f**) show interval volume loss of the bilateral caudate heads, putamina, and peri-insular cortices (*arrows*) associated with residual abnormal signal changes (*asterisks*)

**Fig. 2:**
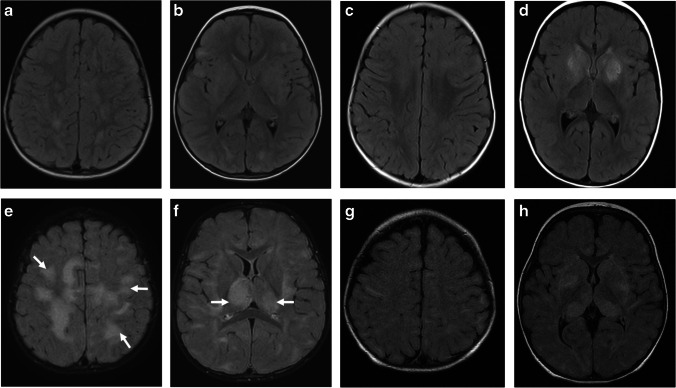
Acute disseminated encephalomyelitis (ADEM) pattern: axial fluid-attenuated inversion recovery (FLAIR) magnetic resonance images of the brain obtained from a 29-month-old boy (**a**, **b**), a 20-month-old girl (**c**,** d**), a 19-month-old boy (**e**,** f**), and a 15-month-old boy (**g**, **h**) with myelin oligodendrocyte glycoprotein antibody seropositivity, who presented with ADEM. Images at the level of the fronto-parietal lobes (**a**, **c**, **e**, **g**) and deep gray matter nuclei (**b**, **d**, **f**, **h**), show varying appearances of involvement of white matter (*arrows* in **e**) and deep gray matter structures (*arrows* in **f**)

On brain MRI, there are multifocal poorly marginated, hazy, patchy, and confluent T2/fluid-attenuated inversion recovery (FLAIR) hyperintensities involving asymmetrically the cerebral white matter and/or gray matter, specifically the juxtacortical white matter and deep gray matter structures. The lesions might be associated with abnormal nodular enhancement. Diffusion restriction is rarely seen, mostly in younger patients, suggestive of cytotoxic edema [14, 18, 33] (Figs. 1 and 2).

**Fig. 3:**
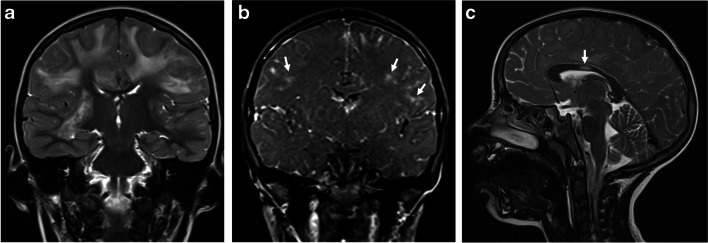
Magnetic resonance images show a leukodystrophy-like pattern in a 4-year-old girl with myelin oligodendrocyte glycoprotein antibody seropositivity, who presented with acute disseminated encephalomyelitis. **a** Coronal T2-weighted image shows bilateral confluent hyperintense signal changes involving asymmetrically the white matter. **b** Coronal fat-suppressed contrast-enhanced T1-weighted image shows bilateral nodular enhancement of the involved white matter (*arrows*). **c** Mid-sagittal T2-weighted image shows a small hyperintense lesion in the body of the corpus callosum (*arrow*)

Younger patients (<5 years) tend to present with larger lesions, including tumefactive lesions (>2 cm). They also tend to have a wider distribution of lesions, including rare involvement of the corpus callosum [14, 34, 35]. The leukodystrophy-like pattern is rarely observed, particularly in very young patients presenting with MOGAD ADEM [22, 36–38]. On imaging, there are extensive confluent and often symmetric T2/FLAIR-hyperintense lesions in the cerebral white matter, associated with nodular enhancement (Fig. 3).

Interestingly, patients presenting with MOGAD ADEM or with MOGAD neuromyelitis optica spectrum disorder-like phenotype exhibit a similar radiological pattern of widespread ADEM-like changes in the brain, despite clinical differences between the two groups [14].

Spinal cord involvement, which may affect up to 75% of MOGAD ADEM patients [14], is an important feature that can be easily overlooked in patients with encephalopathy.

Several publications have attempted to differentiate between MOGAD ADEM and seronegative ADEM based on clinical and radiological findings. They indicate that larger, more diffuse, and bilateral brain lesions, with more frequent involvement of the spinal cord, are more typical of MOGAD ADEM [32]. Nevertheless, currently a reliable differentiation between these two conditions is not possible on a routine daily basis, based only on conventional radiological features.

**Fig. 4:**
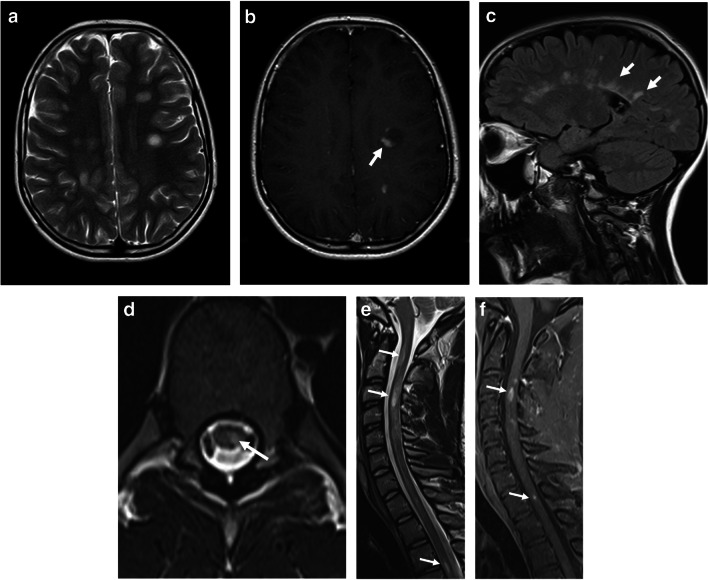
Magnetic resonance images obtained from three different teenagers diagnosed with pediatric-onset multiple sclerosis. **a**–**c** Brain involvement in an 11-year-old girl (**a**, **b**) and a 15-year-old girl (**c**). **a** Axial T2-weighted image shows bilateral, small, well-demarcated ovoid foci of increased T2-signal involving the subcortical and deep fronto-parietal white matter. **b** Axial contrast-enhanced T1-weighted image shows associated open-ring enhancement (*arrow*). **c** Sagittal fluid-attenuated inversion recovery image shows multiple high-signal foci radially oriented, perpendicular to the ventricular wall (*arrows*). **d**–**f** Spinal involvement in a 15-year-old boy. **d** Axial T2-weighted image at thoracic level shows a small hyperintense lesion involving the left lateral aspect of the spinal cord (*arrow*). **e**,** f** Sagittal T2-weighted (**e**) and fat-suppressed contrast-enhanced T1-weighted (**f**) images of the upper spinal cord show short-segment hyperintense T2-lesions (*arrows* in **e**), associated with nodular enhancement (*arrows* in **f**)

The imaging findings in MOGAD ADEM might overlap with POMS and AQP4+NMOSD. Features that are more suggestive of POMS include multiple, well-defined, ovoid T2/FLAIR-hyperintense lesions that asymmetrically involve the periventricular white matter and juxta/intra-cortical regions. Typically POMS lesions abut the ventricular wall perpendicularly (e.g., – Dawson fingers), with frequent involvement of the corpus callosum. Lesions may be associated with ring or open-ring enhancement [7, 33, 39] (Fig. 4). Hypointense T1-weighted lesions (“black holes”) are commonly seen in POMS, reflecting chronicity of disease [40]. Despite earlier publications describing ADEM-like lesions in POMS, it is now clear that these children had MOGAD and were misdiagnosed as multiple sclerosis prior to the antibody discovery. Multiple sclerosis in children looks exactly like multiple sclerosis in adults [23]. Of note, younger age of onset is associated with more inflammatory disease and higher lesion load. In AQP4+NMOSD, brain lesions involve primarily regions with high AQP4 expression: diencephalic regions surrounding the third ventricle and aqueduct, dorsal brainstem abutting the fourth ventricle – specifically the area postrema, and periependymal circumventricular areas. Lesions may be associated with a patchy, cloud-like pattern of enhancement, or with pencil-thin linear periependymal enhancement [7, 41–44].

**Fig. 5:**
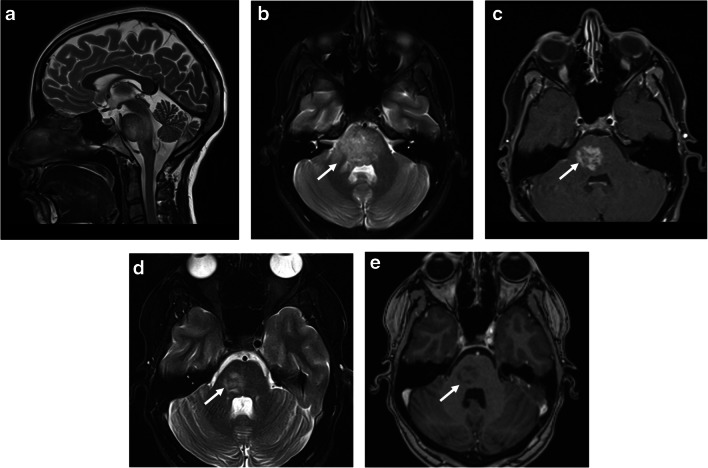
Brainstem involvement in a 17-year-old girl with myelin oligodendrocyte glycoprotein antibody-associated disease, who presented acutely with diplopia, ataxia, and left side hemiparesis. **a** Mid-sagittal T2-weighted, (**b**) axial fat-suppressed T2-weighted, and **c** axial fat-suppressed contrast-enhanced T1-weighted magnetic resonance (MR) images show an ill-defined lesion infiltrating the pons and right middle cerebellar peduncle (*arrow* in **b**) with mass effect on the adjacent fourth ventricle, associated with abnormal enhancement (*arrow* in **c**). **d** Axial fat-suppressed T2-weighted and** e** axial contrast-enhanced T1-weighted MR images obtained at 2-month follow-up show an interval decrease in size of the lesion (*arrow* in **d**), with resolution of enhancement (*arrow* in **e**)

**Fig. 6:**
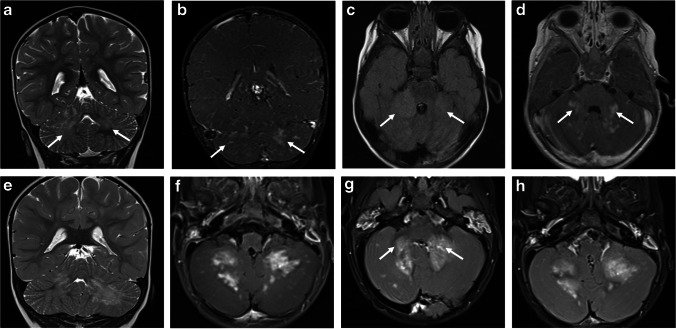
Cerebellar involvement in two children with myelin oligodendrocyte glycoprotein antibody-associated disease and acute disseminated encephalomyelitis. Magnetic resonance images of the brain obtained from a 20-month-old girl (**a**-**d**) and from a 15-month-old girl (**e-h**). **a** Coronal T2-weighted and (**b**) coronal fat-suppressed contrast-enhanced T1-weighted images show ill-defined T2-hyperintensities involving asymmetrically the bilateral cerebellar lobes (*arrows* in **a**), associated with nodular and patchy enhancement (*arrows* in **b**). **c** Axial fluid-attenuated inversion recovery (FLAIR) and (**d**) axial contrast-enhanced T1-weighted images show the lesions involving the bilateral middle cerebellar peduncles (*arrows* in **c** and **d**). **e** Coronal T2-weighted, (**f**) axial fat-suppressed contrast-enhanced T1-weighted, and (**g**, **h**) axial fat-suppressed contrast-enhanced FLAIR images show bilateral lesions involving the cerebellar lobes (**e**) and middle cerebellar peduncles (*arrows* in **g**), associated with avid confluent nodular enhancement (**f**-**h**)

Involvement of the infra-tentorial structures is often associated with MOGAD ADEM, or with other MOGAD phenotypes (transverse myelitis, optic neuritis), seen with variable frequency of 10–67% in mixed pediatric and adult MOGAD cohorts [27, 45]. About two-thirds of these lesions are symptomatic, often manifesting with diplopia and ataxia [27]. Lesions in the region of the area postrema can lead to intractable hiccups, nausea, and vomiting [46]. However, this presentation is more typical of AQP4+NMOSD [15]. MRI findings of brainstem/cerebellar involvement include large ill-defined T2/FLAIR-hyperintensities, mostly involving the pons, unilateral/bilateral middle cerebellar peduncles, and/or cerebellar parenchyma [27, 47] (Figs. 5 and 6). The presence of brainstem lesions that are accompanied by large and ill-defined unilateral/bilateral middle cerebellar peduncle lesions helps in discriminating MOGAD from POMS and AQP4+NMOSD [23, 27, 45, 48, 49]. The cerebellar peduncles contain white matter tracts that are highly myelinated with abundant oligodendrocytes, which may explain the cerebellar involvement in MOGAD [15, 45].

In a retrospective European multicenter study, 5/36 (14%) children presenting with acute cerebellitis were seropositive to MOG antibodies [50]. On MRI, these patients had bilateral (4/5) and unilateral (1/5) involvement of the cerebellum, as well as additional supra- or infra-tentorial lesions. The clinical outcome was generally good.

#### Autoimmune encephalitis

**Fig. 7:**
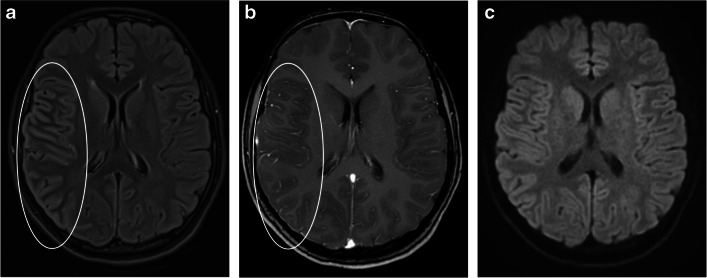
Fluid-attenuated inversion recovery (FLAIR) hyperintense lesions in anti-myelin oligodendrocyte glycoprotein encephalitis with seizures (FLAMES): magnetic resonance images of the brain, obtained from a 14-year-old girl, presented with right ocular symptoms. **a** Axial fat-suppressed FLAIR image shows an increased signal along the right peri-Sylvian and parietal cortex with sulcal effacement (*ellipse*). **b** Axial contrast-enhanced T1-weighted image shows associated leptomeningeal enhancement (*ellipse*). **c** On axial diffusion-weighted image (*b*=1,000), no diffusion restriction is seen. Images courtesy of Dr. Elena Zharkov, Pediatric Radiology Unit, Shaare Zedek Medical Center, Jerusalem, Israel

**Fig. 8:**
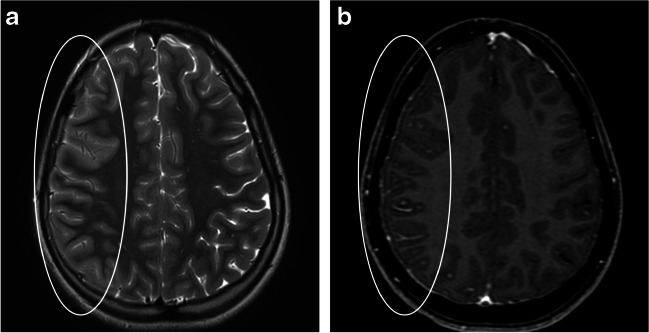
Cortical encephalitis in myelin oligodendrocyte glycoprotein antibody-associated disease. Magnetic resonance images of the brain obtained from a 14-year-old girl. **a** Axial T2-weighted image shows diffuse swelling of the right-sided cerebral cortex with sulcal effacement (*ellipse*). **b** Axial contrast-enhanced T1-weighted image shows asymmetric leptomeningeal enhancement along the involved region (*ellipse*)

**Fig. 9:**
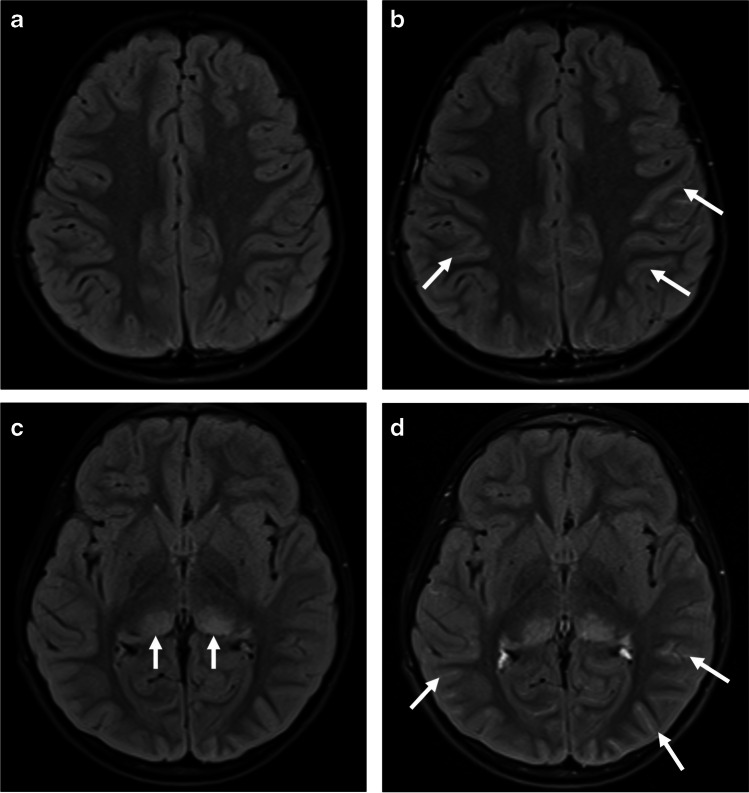
Myelin oligodendrocyte glycoprotein antibody-associated autoimmune encephalitis in a 6-year-old boy, presented with headaches and fever. **a**, **c** Axial fat-suppressed fluid-attenuated inversion recovery (FLAIR) and (**b**, **d**) axial fat-suppressed contrast-enhanced FLAIR magnetic resonance images show bilateral faint leptomeningeal enhancement, more prominent on the left side compared to the right side (*arrows* in **b** and **d**). In addition, there is a symmetric increased signal in the thalami (*arrows* in **c**), but no enhancement (**d**)

This entity was first described in 2017 [51] and is commonly associated with fever, headaches, and seizures. Intra-cranial hypertension can accompany this phenotype [26, 52]. There is a higher prevalence in children, being reported in 12/89 (13.5%) pediatric-onset MOGAD, compared to 7/196 (3.6%) adults with MOGAD [53]. Three main patterns have been described: (1) *Cortical encephalitis* – also termed FLAIR-hyperintense lesions in anti-MOG encephalitis with seizures (FLAMES) [51, 54, 55]. On brain MRI, the T2/FLAIR-hyperintense cortical lesions can be unilateral or bilateral, diffuse, or focal. These lesions can involve the juxtacortical white matter and might be associated with leptomeningeal enhancement and/or diffusion restriction [45, 51, 53, 55] (Fig. 7). Other reported findings include swelling of the cortex with effacement of the sulci [56] (Fig. 8). Unilateral involvement affects more commonly the frontal and parietal lobes, while bilateral involvement affects commonly the frontal lobes. The occipital lobes are rarely involved [22, 55, 56]. (2) *Basal ganglia encephalitis* – radiological findings include bilateral high T2/FLAIR signal involving the basal ganglia and/or thalami [15, 26] (Fig. 9). In a large prospective Spanish study, among 64 patients with autoimmune encephalitis (other than ADEM), MOG antibodies were more common than all other neuronal antibodies combined (33%), even more common than anti-N-methyl-D-aspartate receptor (NMDAR) encephalitis [26]. Of note, in rare cases, MOGAD-encephalitis may coexist either simultaneously or in succession with anti-NMDAR encephalitis [22, 26, 57, 58]. (3) *Meningoencephalitis/aseptic meningitis with leptomeningeal enhancement* – may be seen at presentation of MOGAD in 33–46% of patients [59, 60], even without evidence of demyelination, or may precede demyelination [61], and may be the only radiological finding in MOG antibody-associated aseptic meningitis [55, 62]. On imaging, unilateral or bilateral leptomeningeal enhancement is observed (Fig. 8), and the underlying cortex may be preserved (Fig. 9). In a retrospective cohort of 42 pediatric patients with MOGAD, POMS, and AQP4+NMOSD [63], leptomeningeal enhancement was only seen in MOGAD patients (6/20, 30%). Acquiring contrast-enhanced FLAIR sequences might be helpful for leptomeningeal enhancement detection [64, 65].

The common and/or unique radiological findings involving the brain at initial presentation of MOGAD, POMS, and AQP4+NMOSD are summarized in Table 2.

**Table 2:**
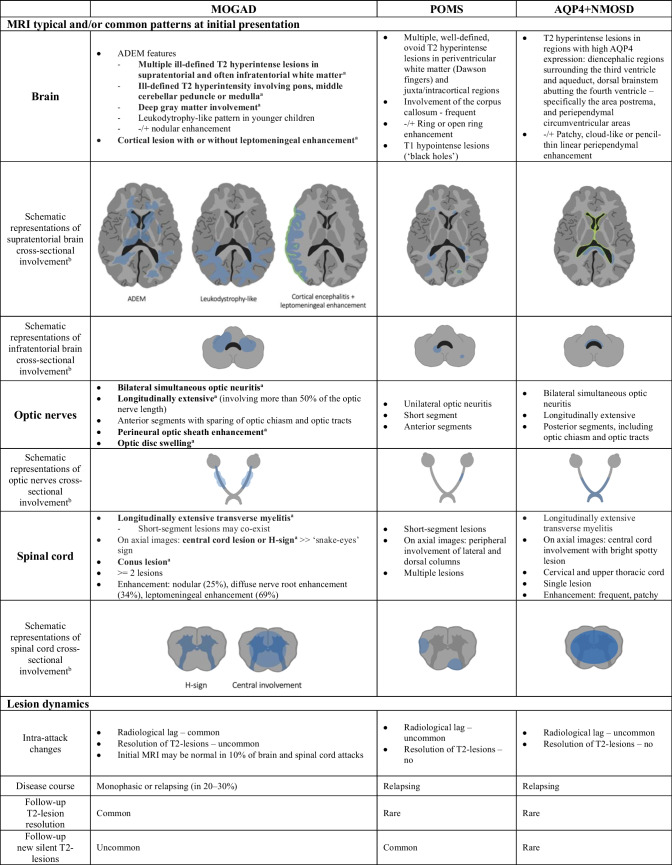
Radiological features and lesion dynamics in pediatric myelin oligodendrocyte glycoprotein antibody-associated disease, pediatric onset multiple sclerosis and aquaporin 4-IgG-seropositive neuromyelitis optica spectrum disorder

^a^MOGAD radiological features that are highlighted in bold are supporting features included in the MOGAD diagnostic criteria [8]

^b^Schematic representations of brain, optic nerves and spinal cord cross-sectional involvement on MRI scans: *blue* represents abnormal T2-hyperintensity, *green* represents abnormal contrast enhancement

*ADEM* acute disseminated encephalomyelitis, *AQP4+NMOSD* aquaporin 4-IgG-seropositive neuromyelitis optica spectrum disorder, *MOGAD* myelin oligodendrocyte glycoprotein antibody-associated disease, *MRI* magnetic resonance imaging, *POMS* pediatric onset multiple sclerosis

### Optic neuritis

MOG antibody seropositivity is reported in 37% of children presenting with optic neuritis [33]. Clinically they may complain of severe new-onset headache, followed by painful eye movements and severe visual deficits at nadir [21]. Often there is optic disc edema, which is a distinguishing feature from POMS and AQP4+NMOSD [66–68].

On images obtained at presentation, bilateral optic nerve involvement is suggestive of MOGAD, being reported with a prevalence between 24–80% in different pediatric or mixed pediatric-adult MOGAD cohorts, and is more prevalent in children compared to adults [24, 66, 67, 69–71].

**Fig. 10:**
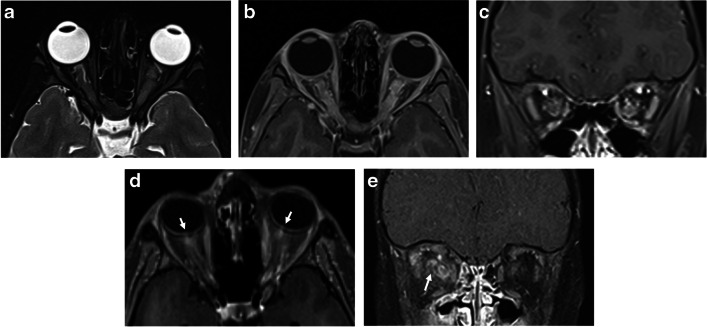
Optic neuritis in three children diagnosed with myelin oligodendrocyte glycoprotein antibody-associated disease. **a**-**c** Magnetic resonance (MR) images of the orbits obtained from a 5-year-old girl presented with encephalopathy and bilateral optic neuritis. **a** Axial fat-suppressed T2-weighted image shows bilateral longitudinally extensive optic neuritis with T2-hyperintensity along the intra-orbital segments of the optic nerves. **b** Axial and **c** coronal fat-suppressed contrast-enhanced T1-weighted images show enhancement along the involved segments, as well as of the surrounding perineural tissues. **d** Axial fat-suppressed contrast-enhanced T1-weighted MR image of the orbits obtained from a 17-year-old girl presented with diplopia and left side paresthesia shows enhancement and swelling of the bilateral optic nerve heads (*arrows*). **e** Coronal fat-suppressed contrast-enhanced T1-weighted MR image of the orbits obtained from a 28-month-old boy presented with encephalopathy shows bilateral asymmetric optic neuritis, more prominent on the right side, with enhancement of the optic nerve, optic nerve sheath, and perineural tissues (*arrow*)

**Fig. 11:**
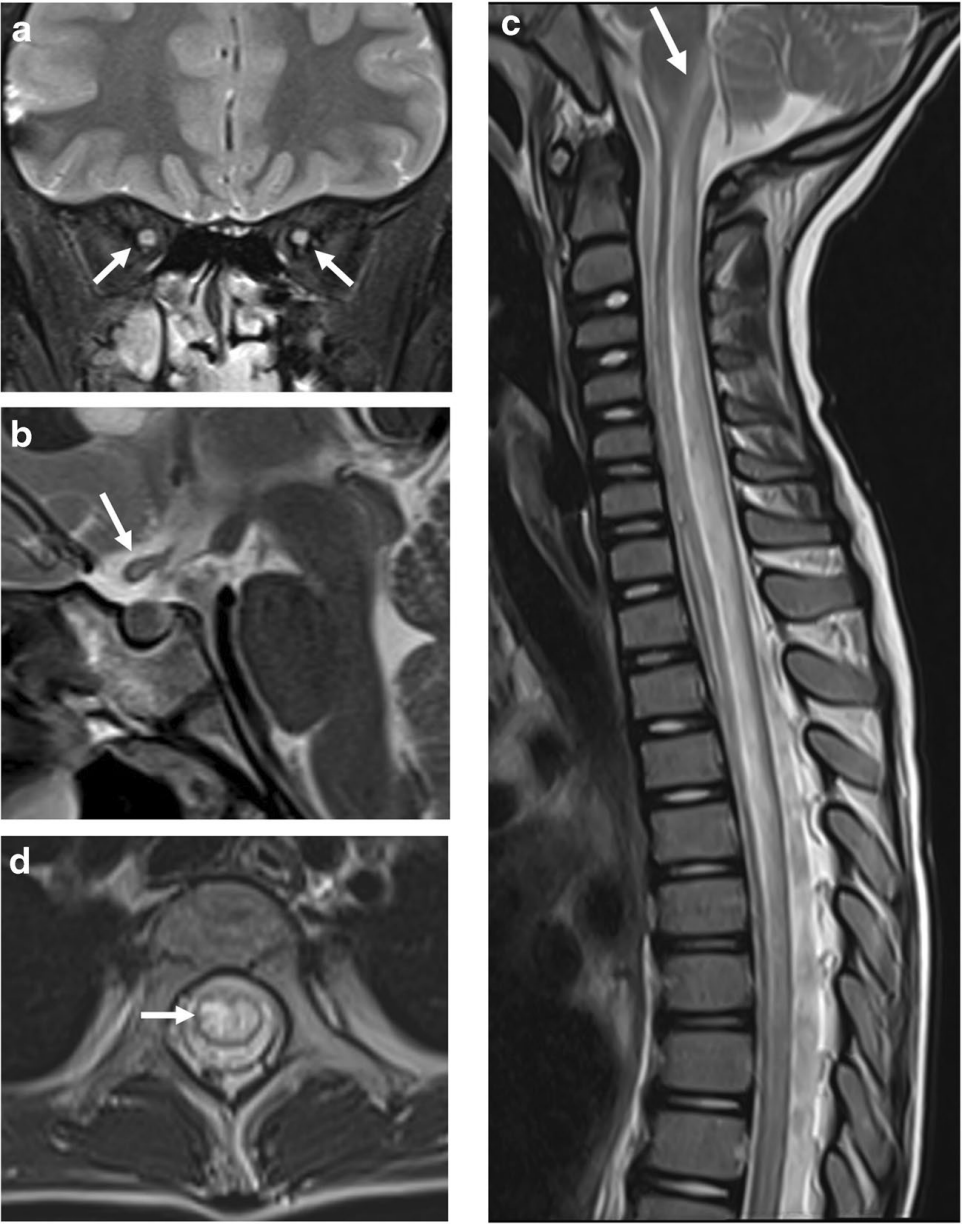
Aquaporin 4-IgG-seropositive neuromyelitis optica spectrum disorder in two children. **a**, **b** Magnetic resonance (MR) images obtained from a 12-year-old girl presented with bilateral optic neuritis. **a** Coronal fat-suppressed T2-weighted image of the orbits shows bilateral high signal involving the proximal intra-orbital optic nerves (*arrows*). **b** Mid-sagittal T2-weighted image of the brain shows a subtle hyperintensity in the optic chiasm (*arrow*). **c**, **d** Spinal involvement in a 5-year-old girl presented with vomiting and paraparesis. **c** Sagittal T2-weighted MR image of the cervico-thoracic cord shows longitudinally extensive transverse myelitis, associated with thickening and hyperintensity along the cervical and upper thoracic cord, extending superiorly to the medulla, specifically towards the area postrema (*arrow*). **d** Axial T2-weighted MR image at the level of the thoracic spinal cord shows high signal involving the central gray matter and surrounding white matter – appearing like a pocket of cerebrospinal fluid, namely the “bright spotty lesion” (*arrow*)

The anterior segments of the optic nerves are typically involved, with relative sparing of the optic chiasm and the optic tracts [24, 72]. The lesions are characteristically longitudinally extensive, involving more than 50% of the nerve length, with T2-hyperintensity and contrast enhancement along the involved nerve. Longitudinally extensive optic neuritis was observed in 16/22 (73%) children with MOGAD and bilateral optic nerve involvement [67] (Fig. 10). Bilateral longitudinally extensive optic neuritis at presentation is characteristic of AQP4+NMOSD as well (Fig. 11), but is not common in POMS [66]. In addition, chiasmatic involvement is much more commonly seen with AQP4+NMOSD (Fig. 11), as compared with MOGAD and POMS [66]. In contrast, optic nerve lesions in POMS are often unilateral and short, with possible enhancement [8, 39, 44, 66].

Perineural enhancement of the optic nerve sheath and surrounding retrobulbar fat is considered a specific finding for MOGAD optic neuritis (Fig. 10), being reported in 25/50 (50%) mixed pediatric and adult patients in a multicenter study [72], and in 17/25 (68%) pediatric patients in a single-center study [69]. However, in another retrospective study of 40 children with MOGAD optic neuritis, a lower prevalence of perineural enhancement was reported, observed in only 6/53 eyes (11.3%) [68]. Optic disc swelling is another typical MOGAD feature, being reported in 10/19 (53%) mixed pediatric and adult cohort [66] (Fig. 10).

The common and/or unique radiological findings involving the optic nerves at initial presentation of MOGAD, POMS, and AQP4+NMOSD are summarized in Table 2.

### Spinal cord involvement

**Fig. 12:**
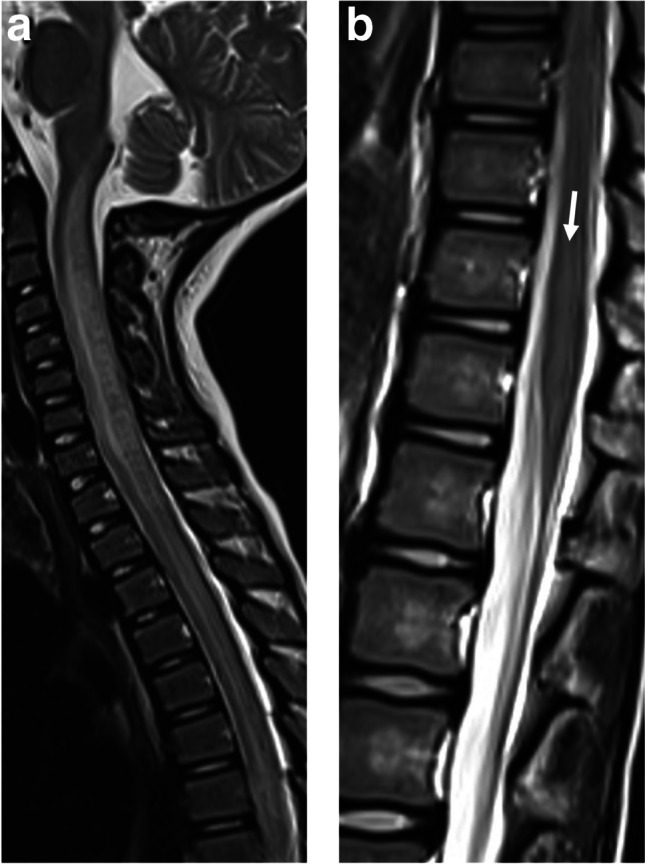
Transverse myelitis in myelin oligodendrocyte glycoprotein antibody-associated disease. **a** Sagittal T2-weighted magnetic resonance (MR) image obtained from a 5-year-old girl shows a longitudinally extensive hyperintensity with swelling along the cervical and upper thoracic cord, extending superiorly to the level of the area postrema. **b** Sagittal T2-weighted MR image obtained from of a 7-year-old girl shows a longitudinally extensive hyperintensity along the lumbar cord, with typical conus involvement (*arrow*)

MOG antibody seropositivity is reported in 13% of children presenting with transverse myelitis at initial attack [33]. Presentation is often severe with paraparesis and sphincter dysfunction, being reported in 45/54 (83%) mixed pediatric and adult MOGAD cohorts [73]. A minority of patients may present with acute flaccid paralysis [73]. The typical radiological involvement is longitudinally extensive transverse myelitis, with T2-hyperintense lesions extending along three or more vertebral segments, being reported in 60–75% of patients in different studies [8, 28, 74] (Fig. 12). Extent of lesions may even span more than ten vertebral segments [75, 76]. The cervical and thoracic cord levels are affected more commonly than the lumbar and conus levels [14, 28, 47, 73, 74]. Extension of cord lesions to posterior medulla oblongata or area postrema region was described in approximately 30% of MOGAD transverse myelitis patients [73, 74] (Fig. 12). Cord swelling of the involved segment may be seen in the acute stage [74]. Shorter lesions may coexist [15], and most patients have at least two separate lesions [73, 74, 77]. More recent studies suggest higher prevalence of short-segment cord lesions than was previously reported [47, 69]. Differences in various reports probably relate to the overall low prevalence of MOGAD transverse myelitis in pediatric populations, resulting in small sample size.

**Fig. 13:**
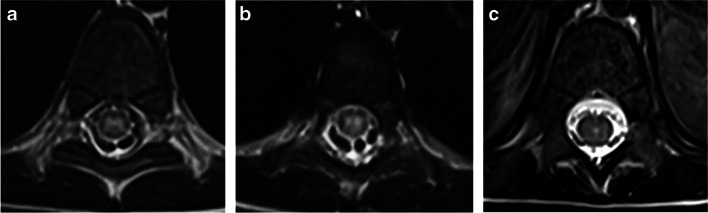
Transverse myelitis in a 4-year-old boy with myelin oligodendrocyte glycoprotein antibody-associated disease. **a**, **b**, **c** Axial T2-weighted magnetic resonance images of the spinal cord show characteristic cross-sectional patterns of increased T2-signal: **a** “H-sign” involving the central gray matter. **b** "Snake-eyes" sign involving the anterior columns. **c** Hazy central involvement of gray and white matter.

Features of spinal cord involvement were assessed by the Canadian Pediatric Demyelinating Disease Study Group in a multicenter study of children with acquired demyelinating syndromes, including 40 MOGAD patients, 21 POMS patients, and 46 seronegative monophasic patients [28]. The cervical cord was the most frequently involved in all three entities. However, involvement of thoracic and conal segments was 1.9 and 2.2 times more common in MOGAD patients as compared to POMS patients. Additional studies supported these findings and reported more frequent involvement of the lumbar and conal regions in MOGAD transverse myelitis, as compared to AQP4+NMOSD, with similar or slightly higher frequency than POMS [73, 75, 77]. Most MOGAD transverse myelitis lesions are centrally located on axial images, involving both gray and white matter, often encompassing the entire cross section (25/35 (71%)) [28]. Lesions may demonstrate prominent gray matter involvement producing the characteristic MOGAD “H-sign” (63%) on axial images, or are restricted to the anterior columns, producing the "snake-eyes" sign (17%) [28] (Fig. 13). In contrast, lesions in POMS transverse myelitis are typically short-segment and peripherally located, involving the lateral and dorsal columns (Fig. 4). Contiguous or confluent POMS lesions may falsely appear as longitudinally extensive disease [7, 8, 39, 44, 66]. In AQP4+NMOSD, the typical spinal cord pattern is longitudinally extensive transverse myelitis, often involving the cervical and upper thoracic cord, with a significant T2-hyperintensity along the central gray matter and associated thickening of the cord (Fig. 11), and with frequent enhancement (patchy or ring like) [41, 75]. The marked T2-hyperintensity often appears like a pocket of CSF, also termed “bright spotty lesion” [7, 41, 42], which is characteristic of AQP4+NMOSD (Fig. 11), and is absent in POMS transverse myelitis and MOGAD transverse myelitis [75]. As opposed to MOGAD transverse myelitis in which several lesions can be observed along the cord, in AQP4+NMOSD, a single longitudinally lesion is seen in the majority of the cases.

**Fig. 14:**
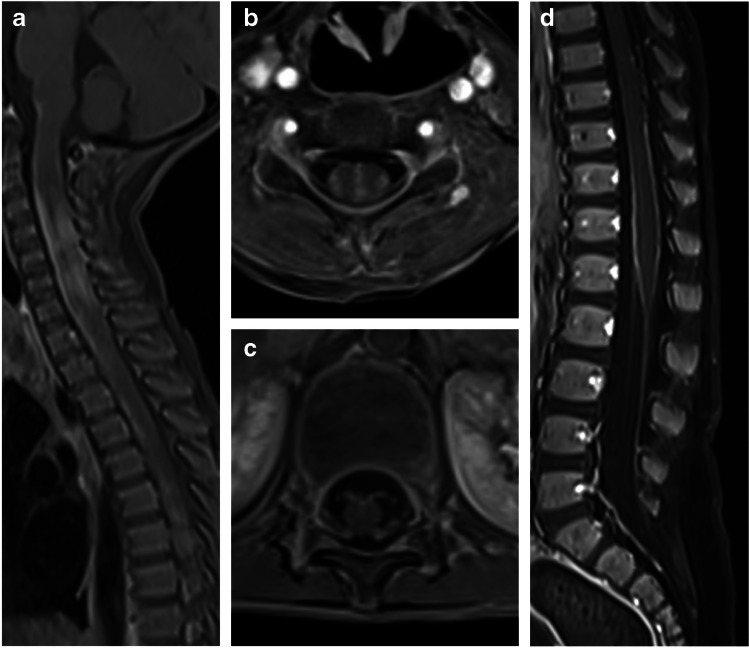
Patterns of enhancement in myelin oligodendrocyte glycoprotein antibody-associated transverse myelitis, on fat-suppressed contrast-enhanced T1-weighted magnetic resonance images obtained from a 5-year-old girl (**a**), a 4-year-old boy (**b**, **c**), and a 21-month-old girl (**d**). **a** Sagittal image shows patchy intra-medullary enhancement along the thickened cervical and upper thoracic cord. **b**,** c** Axial images show intra-medullary and nerve root enhancement at cervical level (**b**), and lumbar nerve root enhancement surrounding the conus (**c**). **d** Sagittal image shows leptomeningeal enhancement along the lower thoracic and lumbar cord

Three patterns of enhancement were observed in 32 pediatric MOGAD transverse myelitis patients: nodular enhancement (25%), leptomeningeal enhancement (69%), and spinal nerve root enhancement (34%) [28] (Fig. 14). Leptomeningeal enhancement was highly suggestive of MOGAD transverse myelitis, compared to POMS and seronegative transverse myelitis [28]. Of note, a single-center study from China [74] described faint and patchy enhancement in 6/14 (43%) MOGAD pediatric patients, and reported leptomeningeal enhancement in 29% and spinal nerve root enhancement in 57% of their cohort.

Spinal cord radiological findings were similar between children with MOGAD ADEM and MOGAD transverse myelitis [28].

The common and/or unique radiological findings involving the spinal cord at initial presentation of MOGAD, POMS, and AQP4+NMOSD are summarized in Table 2.

## Lesion dynamics during and after initial attack

### Intra-attack

**Fig. 15:**
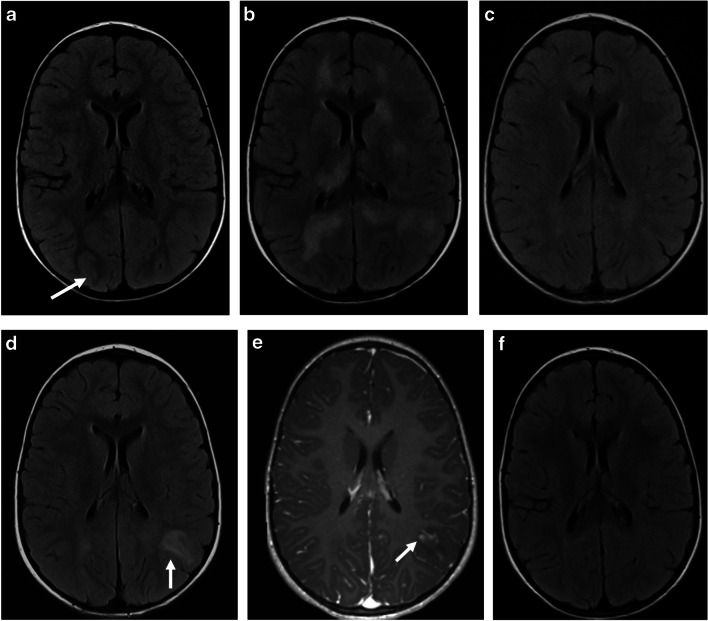
Lesion dynamics in myelin oligodendrocyte glycoprotein antibody-associated disease (MOGAD). **a**-**d**,** f** Axial fluid-attenuated inversion recovery (FLAIR) and (**e**) axial contrast-enhanced T1-weighted magnetic resonance images of the brain, obtained from a 2-year-old boy, who presented with a single focal seizure. **a** MR image at initial presentation shows very subtle subcortical FLAIR hyperintensities (*arrow*). The child developed progressive neurological deterioration a month after initial presentation, including behavioral changes and visual impairment, and was diagnosed with acute disseminated encephalomyelitis (ADEM). MR image obtained at 1-month interval shows characteristic findings of ADEM, including bilateral, diffuse, confluent, and poorly demarcated white matter signal changes distributed asymmetrically with involvement of the basal ganglia and thalami. Serology confirmed myelin oligodendrocyte glycoprotein antibodies. The evolvement of symptoms and radiological findings is typical of MOGAD fluctuation during the same neurological attack. **c** MR image obtained at 6-months post-attack shows a near-complete resolution of the radiological findings. The patient was asymptomatic and his myelin oligodendrocyte glycoprotein antibody titers were negative. **d** MR image obtained at 21-month follow-up shows a new “silent” hyperintense lesion in the left parietal deep and juxta-cortical white matter (*arrow*), (**e**) associated with nodular enhancement (*arrow*). Due to myelin oligodendrocyte glycoprotein antibody seroconversion at that time, the patient started on a monthly maintenance therapy. **f** MR image obtained at 30-month post-attack shows interval resolution of the “silent” lesion shown on (**d**, **e**)

**Fig. 16:**
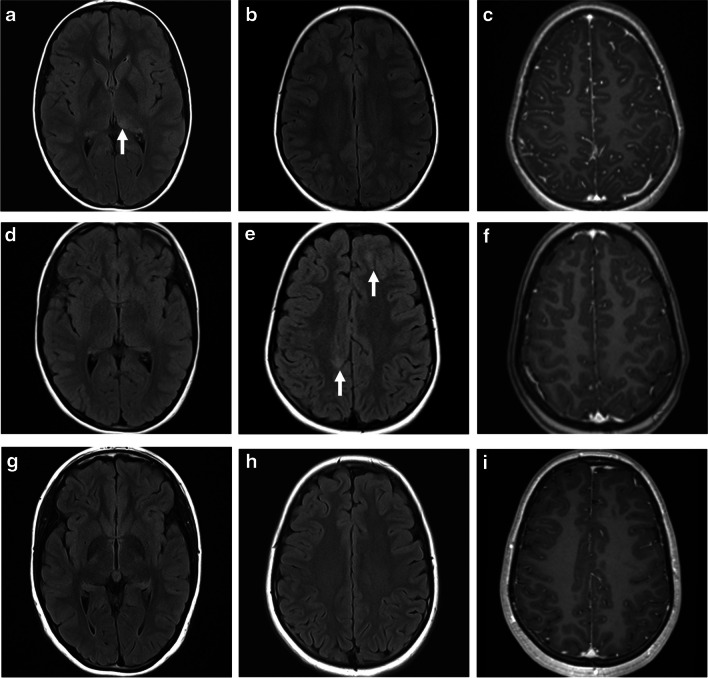
Lesion dynamics in myelin oligodendrocyte glycoprotein antibody-associated disease. **a**-**c** Magnetic resonance images of the brain obtained from an 11-year-old girl who presented with headache and vomiting, and was seropositive to myelin oligodendrocyte glycoprotein antibodies. **a**, **b** Axial fluid-attenuated inversion recovery (FLAIR) images show focal increased signal in the left thalamus (*arrow* in **a**), with diffuse sulcal effacement (**b**). **c** Axial contrast-enhanced T1-weighted image shows bilateral leptomeningeal enhancement. **d**-**f** MR images obtained at 3-month post-attack. **d**, **e** Axial FLAIR images show interval resolution of the signal abnormality in the left thalamus (**d**), with normal appearing sulci (**e**). **f** Axial contrast-enhanced T1-weighted image shows interval resolution of leptomeningeal enhancement. However, there are new subcortical increased FLAIR signal lesions (*arrows* in **e**) that are likely representing radiological lag phenomenon, as the patient was asymptomatic at that time. **g**, **h** Axial FLAIR and (**i**) axial contrast-enhanced T1-weighted images, obtained at 4-year post-attack, show normal appearance of the brain. This patient did not receive maintenance therapy

Timing of imaging is crucial in MOGAD, with different appearances according to the disease stage – acute, relapse, or follow-up. Clinical signs and MRI findings can evolve and fluctuate during disease attack, up to 3 months after onset of ADEM presentation [78], and up to 1 month after onset of other MOGAD presentations [8] (Figs. 15 and 16). This phenomenon is regarded as “radiological lag” of disease [10] and does not reflect a relapse; hence, establishing an accurate phenotypic diagnosis might be challenging during the initial presentation period. MRI scans can be normal at presentation, as have been reported in up to 10% of spine scans of transverse myelitis patients [28, 79]. In a recent retrospective multicenter study, normal brain MRI was observed in 6/58 (10%) MOGAD patients with cerebral symptoms [10]; all of them had abnormalities detected on a second scan acquired after a median of 10 days from the initial study (IQR 7.5–15.5). Therefore, if clinical signs persist in the presence of a normal MRI, a repeat scan within several days is suggested [15, 17, 21, 22, 79]. Intra-attack brain lesion dynamics were assessed on consecutive MRI scans of 58 patients, acquired within a median of 8 days [IQR 5–13] [10]. Appearance of new T2-lesions was observed in 47%, resolution of lesions in 7%, or both new and resolved T2-lesions in 5%. Similar findings were seen in pediatric and adult patients, suggesting a disease-associated biologic mechanism, and not an age-dependent process.

### Clinical relapse

Most pediatric MOGAD presentations are monophasic; however, 20–30% of children may exhibit a relapsing disease [14, 16, 26, 80, 81]. The time to first relapse is commonly within 1 year and typically within 3 years; however, first relapse events were documented even more than 10 years after initial attack [81]. Younger children (<10 years) tend to present with severe disability at disease onset, but frequently demonstrate substantial recovery as compared to older patients, with lower relapse rate [14, 34]. Published data regarding risk factors for relapsing course is inconclusive. Several studies reported increased risk associated with persistent MOG antibody seropositivity or with high MOG antibody titers [26, 34]. Nevertheless, other publications did not observe such associations [69, 82], and relapse of disease occurred also in patients who converted to seronegativity [20]. No radiological features at onset have been associated with risk of relapse [69, 80], but worsening of imaging with new lesions appearing at first follow-up MRI is a predictor of relapse [11].

**Fig. 17:**
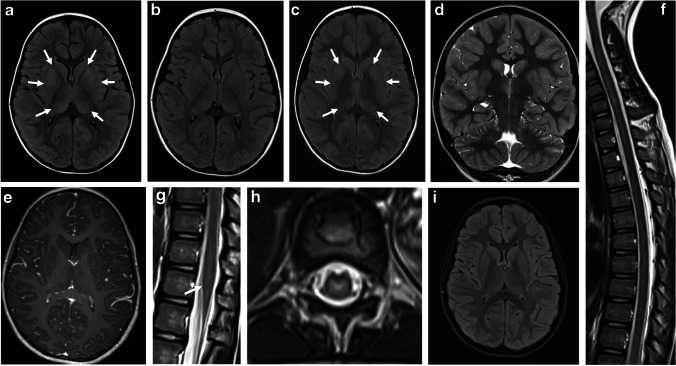
Lesion dynamics in myelin oligodendrocyte glycoprotein antibody-associated disease. **a** Axial fluid-attenuated inversion recovery (FLAIR) magnetic resonance image obtained from a 4-year-old girl who presented with encephalopathy shows mild swelling and increased symmetric signal in the deep gray nuclei (*arrows*). **b** Axial FLAIR MR image at 3-month follow-up shows interval resolution of the radiological findings. **d**-**h** MR images obtained during first clinical relapse of disease, at the age of 7-years (4 years after initial attack). The girl presented with encephalopathy, urinary incontinence, and leg weakness. **c** Axial FLAIR and (**d**) coronal T2-weighted MR images show global cortical swelling with sulcal effacement, and nearly symmetric signal changes in the basal ganglia and thalami (*arrows* in **c**). **e** Axial contrast-enhanced T1-weighted MR image shows diffuse leptomeningeal enhancement. **f**, **g** Sagittal T2-weighted MR images of the spine show an extensive abnormal hyperintensity involving almost the entire cord, including the conus (*arrow* in **g**), consistent with longitudinally extensive transverse myelitis. **h** Axial T2-weighted MR image shows central hazy involvement of the conus. During disease relapse, the patient was myelin oligodendrocyte glycoprotein antibody seropositive and started on a maintenance therapy. **i** Axial FLAIR MR image obtained at 12-months follow-up after disease relapse shows interval resolution of the cerebral signal changes

The clinical-MRI phenotype at relapse may differ from the phenotype at initial presentation, and different patterns may evolve during the disease course [81]. The most common variation is the development of optic neuritis at subsequent relapse. Other relapse patterns include multiphasic disseminated encephalomyelitis, ADEM-optic neuritis, transverse myelitis, neuromyelitis optica spectrum disorder-like phenotype, autoimmune encephalitis, leukoencephalopathy-like pattern, and other unclassified presentations [36, 81] (Fig. 17). In a single-center retrospective study of 61 children with MOGAD, age-related phenotype persisted into first relapse (but not into second relapse), with younger children (≤9 years) most commonly presenting with ADEM-like pattern (34.8%), and older children (>9 years) most commonly presenting with FLAMES at first relapse [69]. In a prospective multicenter study of 102 children with MOGAD, first relapse phenotype demonstrated age dependency, with multiphasic disseminated encephalomyelitis and ADEM-optic neuritis more prevalent in children ≤9 years (Fig. 17), and recurrent optic neuritis and neuromyelitis optica spectrum disorder-like phenotype more prevalent in children older than 9 years [83].

### Follow-up – lesion resolution and silent lesions

**Fig. 18:**
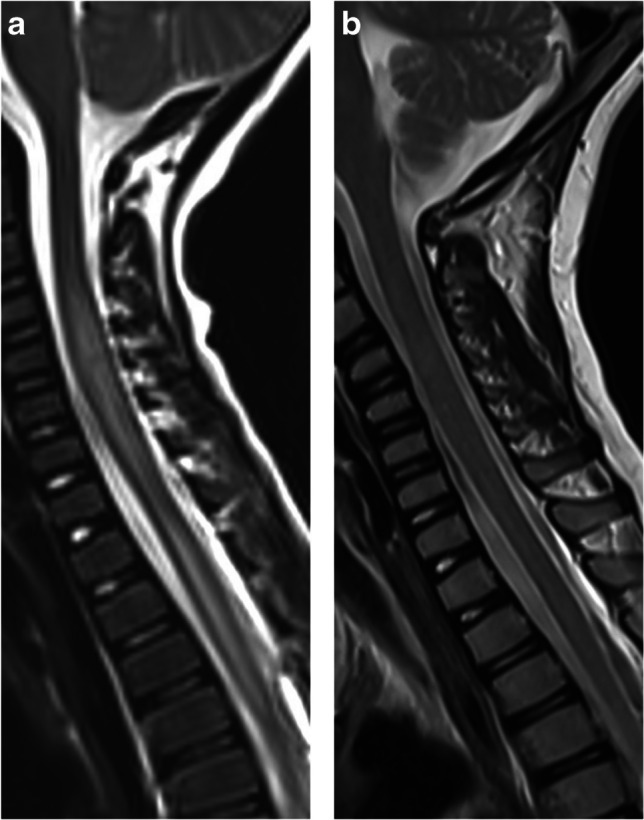
Lesion dynamics in a 4-year-old male with myelin oligodendrocyte glycoprotein antibody-associated disease (MOGAD). **a**, **b** Sagittal T2-weighted magnetic resonance images obtained from a spinal scan during attack (**a**) and at 1-month follow-up (**b**) show typical MOGAD interval complete resolution of the abnormal T2-hyperintensity along the cervical and upper thoracic cord

Recent publications have addressed post-attack evolution of T2-lesions overtime in brain and spine follow-up MRI scans in MOGAD patients. Two main features have been described: resolution of previously documented attack-related T2-lesions, and interval appearance of new T2-lesions. Post-attack resolution of the clinical symptoms and radiological T2-lesions is expected in approximately 60–83% of MOGAD patients (pediatric or mixed pediatric and adult cohorts) [9, 20, 26, 32, 84] (Figs. 15–18). In a recent retrospective multicenter UK study of 97 children with MOGAD, 83% had at least one lesion resolved at first follow-up study, as opposed to only 1/103 (1%) child with POMS, who had a single lesion disappeared [11]. In a retrospective single-center study, features of T2-lesion evolution over time were assessed in pediatric MOGAD cohort, as compared with children with AQP4+NMOSD and POMS [85]. They included altogether 56 patients with 69 attacks. T2-lesions resolved more frequently in MOGAD (brain 9/15 (60%) and spinal cord 8/12 (67%)), than in AQP4+NMOSD (brain 1/4 (25%) and spinal cord 0/7 (0%)), and POMS (brain 0/18 (0%) and spinal cord 1/13 (8%)), *P*<0.01. Other studies demonstrated similar trends of MOGAD T2-lesion resolution in brain and spinal cord scans [28, 76, 84]. Thus, T2-lesion resolution overtime favors MOGAD diagnosis, over AQP4+NMOSD and POMS.

**Fig. 19:**
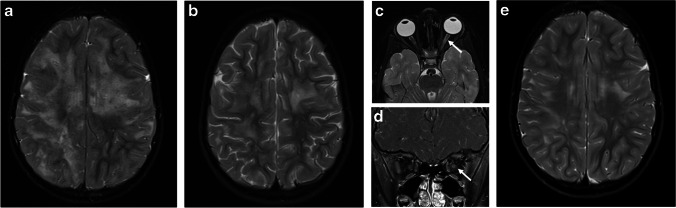
Lesion dynamics in myelin oligodendrocyte glycoprotein antibody-associated disease: evolution of leukodystrophy-like pattern in a 4-year-old girl (same patient as in Fig. 3). **a**-**e** Magnetic resonance (MR) images obtained at initial presentation (**a**), at 1-month follow-up (**b**-**d**), and at 2-year follow-up (**e**). **a**, **b**, **e** Axial fat-suppressed T2-weighted images show interval decrease in size and extent of bilateral diffuse T2-hyperintensities involving the cerebral white matter. However, residual signal abnormalities are evident. The girl did not have a clinical relapse of disease in the 2-year follow-up. **c** Axial fat-suppressed T2-weighted and (**d**) coronal fat-suppressed contrast-enhanced T1-weighted MR images of the orbits obtained at 1-month follow-up show left-sided longitudinally extensive optic neuritis with T2-hyperintensity (**c**) and faint enhancement (**d**). The relatively mild involvement of the left optic nerve and lack of involvement of the right optic nerve might be attributed to treatment response, and the findings were considered as part of the initial attack

Timing and predictors of T2-lesion resolution were assessed in a total of 583 brain and spinal cord lesions (55 pediatric and adult patients) [9]. The median time to resolution of a T2-lesion was 3 months (IQR 1.4–7.0), and most lesions resolved within the first 12 months. Nearly 60% of T2-lesions resolved in the absence of treatment. However, acute T1-hypointensity associated with T2-lesions decreased the likelihood of resolution (odds ratio [95%]). Persistent T2-lesions might represent a higher degree of parenchymal destruction and axonal loss, beyond repair and remyelination capacity [9] (Figs. 1, 5, and 19). In a retrospective multicenter UK study [11], the proportion of normal brain MRI scans was reduced after each clinical relapse, with a marked reduction of the ability of brain repair after the third clinical attack. Early treatment with steroids and/or plasma exchange improves T2-lesion resolution [9, 11].

Interval appearance of new silent T2-lesions has been reported in 10/74 (14%) brain scans of children with MOGAD [86] (Fig. 15), often within the first months after onset of disease, with a low positive predictive value (20%) for relapsing disease. Similar trends with rare appearances of new or enlarging lesions outside of attacks were reported by other groups [87, 88]. However, Camera et al. [87] observed that such lesions appear to indicate a high risk of imminent relapse. In a retrospective multicenter UK study [11], new symptomatic and asymptomatic lesions were found in 39/97 (40%) children at first follow-up scans and were associated with increased risk for relapse, as described above.

Conflicting results regarding the association between appearance of interval silent lesions and risk for relapse may be attributed to the timing of follow-up scans. Previous studies demonstrated variabilities regarding the time lag between attack MRI scan and follow-up MRI scan, and whether the follow-up MRI scan was acquired during remission or at relapse of disease. Lesions may accumulate during a prior clinical attack; thus, an early after-attack MRI scan may not truly reflect interval appearance of new T2-lesions.

Until recently, patients with MOGAD were thought to have a favorable outcome, compared to patients with POMS and AQP4+NMOSD, with good long-term functional outcomes, in keeping with the radiological resolution. Nevertheless, the effect of MOGAD on brain growth trajectories was recently quantified by longitudinal volumetric analysis of brain structures in a cohort of 46 childhood-onset patients. Reduced age-expected growth of deep gray matter structures was observed (*P*<0.001), with a steeper divergence in the first year post onset of disease, that was detected also in children with monophasic course of disease [89]. Poor outcome has been associated with two clinical-MRI progressive entities: (1) The leukodystrophy-like phenotype typically presents initially as ADEM. However, some patients will develop this imaging pattern following multiple attacks and when occurs, lesions are less likely to disappear and are typically associated with worse prognosis and progressive bilateral white matter changes [26, 36]. (2) Cortical/basal ganglia encephalitis that in some patients may be associated with increased intra-cranial hypertension and progressive evolution of severe brain atrophy [26].

In Table 2, there is a summary of lesion dynamics in pediatric MOGAD, compared to POMS and AQP4+NMOSD.

## Summary and future directions

The phenotypic clinical-MRI spectrum of MOGAD is broad, with continuous recognition of new associated radiological patterns and characteristic lesion dynamics. Imaging has an essential role in the recently published international diagnostic MOGAD criteria, and the radiologist should be familiar with the described supporting imaging features. Early identification of patterns that are associated with poor prognosis or with high relapse rate may affect treatment decisions and surveillance. MOGAD has been increasingly recognized as a dynamic disease, with intra- and post-attack evolution of lesions appearing and disappearing, a highly discriminating feature from POMS and AQP4+NMOSD. Initial MRI might be normal in 10% of spine and brain attacks, which might be a caveat to the newly diagnostic criteria. Awareness of such a possibility should prompt a repeat scan if clinical symptoms persist.

Advanced imaging techniques may further improve ability to differentiate between pediatric MOGAD, POMS, and AQP4+NMOSD. Susceptibility-based imaging-related signs have been assessed in pediatric cohorts for detection of central vein sign (CVS), paramagnetic rim lesions, and central core lesions, features that are considered highly specific for adult-onset multiple sclerosis [90, 91]. Sacco et al. [90] reported the prevalence of central vein sign positive rate as a distinguishing feature between pediatric MOGAD and POMS, with ROC=1, *P*<0.0001 (cutoff 41%). Paramagnetic rim lesions were only detectable in POMS patients. Other studies assessed volumetric analysis and diffusion tensor imaging (fractional anisotropy and mean diffusivity) mostly in adult acquired demyelinating disease [92–94]. Future studies assessing advanced techniques in pediatric MOGAD may shed a light on pathobiology of disease and may provide information related to microstructural changes and white matter integrity in different age groups, and in comparison to POMS and AQP4+NMOSD.

Further multicenter large-cohort studies are needed to validate more solid risk factors for relapse and adverse course of MOGAD. Establishing standardized MRI protocols with regular scanning interval may be of aid. We suggest to acquire a follow-up MRI scan 3 months after initial attack, as a new baseline and for assessment of radiological lag features. Currently, the diagnosis of relapsing MOGAD is restricted to patients with clinically relapsing disease and is not based on imaging alone [8]. Considering the rarity of interval new lesions, the value of routine MRI surveillance is questionable and necessitates further research. However, surveillance protocols should be individually tailored, and follow-up MRI every 6 months is recommended for patients with relapsing disease on maintenance treatment.
